# Risk factors associated with football injury among male players from a specific academy in Ghana: a pilot study

**DOI:** 10.1038/s41598-023-34826-0

**Published:** 2023-05-18

**Authors:** Samuel Koranteng Kwakye, Karien Mostert, Daniel Garnett, Andries Masenge

**Affiliations:** 1 Department of Physiotherapy, Faculty of Health Sciences, University of Pretoria, Pretoria, South Africa; 2 Department of Sport, Health Sciences and Social Work, Faculty of Health and Life Sciences, Oxford Brookes University, Oxford, UK; 3 Department of Statistics, University of Pretoria, Pretoria, South Africa

**Keywords:** Health care, Risk factors

## Abstract

There seems to be no information on the incidence of injury and associated risk factors for academy football players in Ghana. We determine the risk factors associated with match and training injuries among male football players at an academy in Ghana. Preseason measurements of players’ height, weight, and ankle dorsiflexion (DF) range of motion (ROM) were measured with a stadiometer (Seca 213), a digital weighing scale (Omron HN-289), and tape measure, respectively. The functional ankle instability (FAI) of players was measured using the Cumberland Ankle Instability Tool (CAIT), and dynamic postural control was measured with the Star Excursion Balance Test. Injury surveillance data for all injuries were collected by resident physiotherapists throughout one season. Selected factors associated with injury incidence were tested using Spearman’s rank correlation at a 5% significance level. Age was negatively associated with overall injury incidence (r = − 0.589, *p* = 0.000), match (r = − 0.294, *p* = 0.008), and training incidence (r = − 0.314, *p* = 0.005). Previous injury of U18s was associated with training injuries (r = 0.436, *p* = 0.023). Body mass index (BMI) was negatively associated with overall injury incidence (r = − 0.513, *p* = 0.000), and training incidence (r = − 0.395, *p* = 0.000). CAIT scores were associated with overall injury incidence (n = 0.263, *p* = 0.019) and match incidence (r = 0.263, *p* = 0.029). The goalkeeper position was associated with match incidence (r = 0.241, *p* = 0.031) while the U16 attacker position was associated with training incidence. Exposure hours was negatively associated with overall injury incidence (r = − 0.599, *p* = 0.000). Age, BMI, previous injury, goalkeeper and attacker positions, ankle DF ROM, and self-reported FAI were associated with injury incidence among academy football players in Ghana.

## Introduction

Football injuries and risk factors have been reported among professional, elite adult, and youth players^1,2^. Relatively few studies have focused on elite players between 16 and 32 years in Africa^3,4^. Currently, emphasis is placed on reducing injury prevalence and incidence, improving performance, and extending athletes' active lifespan^5,6^. Recent research has shown that preventing sports injuries can improve the long-term health and wellness of players^7,8^. Reduced ankle dorsiflexion (DF) range of motion (ROM) may increase the risk of certain lower extremity injuries by modifying the stiffness of the leg and landing forces after a jump^9^. Reduced ankle DF ROM has been shown to be associated with ankle injuries, hamstring injuries, Achilles tendon injuries, patellar tendon injuries, and anterior cruciate ligament (ACL) tears^10–15^. Intrinsic factors, such as age and previous injuries, have also been shown to be associated with hamstring injuries^14^. Proprioception has been associated with ankle injuries^16^, and limb dominance has been associated with ACL injuries^17^. Dynamic postural control has been associated with lower extremity injuries^18^. Extrinsic factors that have been shown to be associated with lower extremity football injuries in some studies include exposure time, player position, and being a member of a national team^19,20^.

Risk factor analysis in any sport is important for preventing injuries per the van Mechelen model^21^. Understanding the trends of injury and associated risk factors, regardless of the level of involvement, may help stakeholders establish effective measures to mitigate injuries^22^. Prospective studies identifying risk factors associated with football injuries among male academy football players are scarce worldwide, including Africa. Only a few retrospective studies have been conducted on Ghanaian football injury and its risk factors, and the validity of these studies may be influenced by possible misclassification or recall bias. Specific injury prevention programs are developed per injury rates, injury types, and anatomical sites among other injury characteristics and environmental factors^23^. These factors appear to differ according to the contexts of players^1,2^. In the Ghanaian region, little is known about the context of academy football players, thus more information is needed to plan and implement successful injury prevention programs. Moreover, translating injury prevention concepts into actual practice involves having a thorough understanding of the individual, social, environmental, and sporting delivery aspects^23^. To the best of our knowledge, this is the first prospective study assessing the incidence of football injuries and associated risk factors among Ghanaian academy footballers. Additionally, the academy players who participated in the study trained and competed primarily on artificial turf, which is uncommon in Africa, particularly Ghana. With the increasing number of budding football academies and the installation of artificial turfs in Ghana, it is imperative to assess the risk factors associated with football injuries among different age groups of footballers over a season to implement optimum training strategies. The aim of the study, therefore, was to determine the risk factors associated with football injuries among youth and adult football players at an academy in Ghana.

## Methods

### Study design and participants

An observational prospective cohort study was conducted among 80 male players at a football academy in Ghana. In addition to the informed consent provided by each participant, players under the age of 18 also gave their assent and their parents' consent. Participants included male youth and adult football players who were enrolled into the football academy. All players who were scheduled to be transferred to other teams, or players whose contracts were set to expire in the month following data collection were excluded from the study. Players were grouped into four teams according to their age, which included U14 (< 14 years), U16 (< 16 years), U18 (< 18 years), and a senior team (≥ 18 years). The football academy situated in the Volta region of Ghana comprises three standard artificial turfs, a physiotherapy unit, one gymnasium, and accommodation for all players and some staff. The academy enrolled only male football players, who stay in the football camp for approximately ten months each year and are given a special diet three times a day, monitored sleep by supervisors, attend a government school, and have access to physiotherapists every day. In a week, training involved five to seven training sessions for all age groups with each session spanning between 90 and 120 min. The senior team, in addition, have one strength, agility and quickness (SAQ) session (30 min); one power training session (30 min); and one injury prevention session (45 min) per week. The U18 players have one SAQ session (30 min) and one injury prevention session (45 min) per week. The U14 and U16 players do not have specific training sessions.

### Data collection

Before the start of the season, two resident physiotherapists who worked at the academy administered a questionnaire to collect demographic and injury information, including current injury status, previous injury (defined as any injury sustained by a player and was diagnosed by the resident physiotherapists and recorded in their notes in the last 12 months), age, player position, dominant leg, and being a member of a national team. Selected risk factors that were investigated included previous injury, age, dominant leg, ankle dorsiflexion (DF) range of motion (ROM), functional ankle instability (FAI), dynamic postural control, body mass index (BMI), player position, being a member of a national team, match and training exposure hours.

Preseason measurements of height, weight, and ankle dorsiflexion ROM were measured and recorded on a standardised injury surveillance (SIS) form by the primary researcher and two trained physiotherapists using a stadiometer (Seca 213), a digital weighing scale (Omron HN-289), and a tape measure, respectively. FAI was assessed using the Cumberland ankle instability tool (CAIT). The CAIT is a valid and reliable questionnaire for measuring subjective FAI with an outstanding test–retest efficiency of 0.96 intra-class correlation coefficient (ICC)^24^. The questionnaire took approximately 7 min to complete. For the CAIT, the cut off for self-reported FAI was 27.5, with FAI ≤ 27.5 indicating ankle instability^24^. The Star Excursion Balance Test (SEBT) was used to assess dynamic postural control. The SEBT has demonstrated excellent reliability (ICC = 0.89–0.94) for the measurement of dynamic postural control^25^. The composite score of the SEBT was calculated as the sum of the maximum score for reach distances divided by three times the limb length and multiplied by 100^28^. The procedure of SEBT was completed as described by Ness et al.^25^. All procedures were performed in accordance with relevant guidelines and regulations.

The injury surveillance period began after the preseason measurements had been completed in August 2021 and the season started in September 2021. The season lasted for 39 weeks, from September 2021 to June 2022. The physiotherapists were trained on how to record injuries on the injury surveillance form according to the International Olympic Committee (IOC) guidelines^26^ over the season. The physiotherapists were always present at training and matches, and they identified injuries as and when they occurred or when they were reported by players on the SIS form. Injury data collection included multiple categories of information but for the purpose of this study, only the occurrence of a ‘medical attention’ injury (an injury that results in the player seeking medical attention from the physiotherapist or causing the player to be absent from the next match or training) was included in the analysis. Weekly records (in min) of each player's exposure during training and matches were recorded on an exposure form by the assistant coaches after every match or training session on the field.

Confounding variables were minimized because all players, with the exception of the senior team, played all their games on artificial turf. Additionally, training styles were the same for each age group as coaches and technical personnel remained unchanged throughout the season. In addition, the environment or climate for each player was comparable because all training and matches were held at the academy in Ghana's Volta Region, with the exception of the senior team, which participated in a few matches in other parts of the country.

### Data analysis

The SPSS IBM Statistics version 28 software was used to perform the analysis. Injury incidence was calculated per 1000 exposure hours. The data analysis consisted of descriptive statistics (means, median, and standard deviations) for continuous variables (BMI, ankle DF ROM, SEBT scores, CAIT scores and exposure hours) and frequency tables (counts and percentages) for categorical variables (age, limb dominance, previous injury, player position, national team membership) to describe the characteristics of the population. The non-parametric Spearman’s rank correlation was performed to determine if the selected factors were associated with injury incidence. The Spearman rank correlation was appropriate because the following extrinsic factors (player position, national team member) and intrinsic factors (previous injury, limb dominance) were dichotomised to have a value of 0 or 1, while the dummy variables were created for the factor position of the player, and some of the continuous data were not normally distributed. The Spearman rank correlation coefficients range between − 1 and 1, with values close to − 1 indicating a negative perfect relationship and values close to 1 indicating a positive perfect relationship. Results were interpreted at a significance level of 5% (*p* < 0.05).

### Ethical approval and consent

Ethics approval was granted by the Ethics Committee of the Faculty of Health Sciences at the University of Pretoria (Reference no. 268/2021). All procedures were performed in accordance with relevant guidelines and regulations. Adult players gave informed consent while players below 18 years gave assent and parents’ consent before participating in the study. Informed consent was obtained from all participants and/or their legal guardian(s).

## Results

### Demographic and player-specific factors

The population of players at the academy for the 2021/2022 season was 105. All players were invited to participate, but only 80 (76%) players participated in the study based on inclusion criteria, willingness to participate, and obtaining of consent. Out of the 105 players who were present at the academy during the time of data collection period, a total of 25 players were excluded from the study. Notably, this included 15 trial players, five players who were in the process of being transferred to another team, three young players who could not obtain parental consent, and two players whose contracts were scheduled to end within the following month. Table 1 shows the demographic and player-specific variables for the total population and the different age groups.

**Table 1:** Description of population by demographic and player-specific variables [n = 80].

Variable	Total population [n = 80]	U14 [n = 7]	U16 [n = 17]	U18 [n = 29]	Senior Team [n = 27]
Age [mean, SD]	17, ± 3.10	13, ± 0.38	15, ± 0.51	17, ± 0.51	21, ± 2.65
BMI [mean, SD]	20.14, ± 2.19	17.6, ± 2.0	18.47, ± 1.21	20.28, ± 1.87	21.69, ± 1.71
Previous injury					
Yes [n, %]	63, 78.8	5, 71.4	12, 70.6	25, 86.2	21, 77.8
No [n, %]	17, 21.3	2, 28.6	5, 29.4	4, 13.8	6, 22.2
Limb dominance					
Left [n, %]	13, 16.3	1, 14.2	0.0	6, 20.7	6, 22.2
Right [n, %]	67, 83.8	6, 85.7	17, 100	23, 79.3	77.8
Member of a national team					
Yes [n, %]	9, 11.3	0, 0.0	0.0	3, 10.3	6, 22.2
No [n, %]	71, 88.8	7, 100	17, 100	26, 89.7	21, 77.8
Player position					
Goalkeeper [n, %]	9, 11.3	0, 0.0	3, 17.7	3, 10.3	3, 11.1
Defender [n, %]	29, 36.6	2, 28.6	7, 41.2	11, 37.9	9, 33
Midfielder [n, %]	19, 23.8	3, 42.9	3, 17.7	5, 17.2	8, 29.6
Attacker [n, %]	23, 28.8	2, 28.6	4, 23.5	10, 34.5	7, 25.9

### Injury incidence

There were 126 injuries in total, most of which were acute (n = 97, 77%), while only 29 (23%) were overuse injuries. About 65 (51.6%) injuries involved contact, while 61 (48.4%) injuries were non-contact injuries. The most frequently injured body site was the knee (n = 30, 23.8%), and the most frequent injury type was joint sprain (n = 54, 42.9%). The overall injury incidence rate was 4.5 injuries per 1 000 exposure hours. Table 2 shows the injury incidence rates per age group.

**Table 2:** Injury incidence rate among male football players at a Ghanaian football academy.

	Overall incidence			Match incidence			Training incidence		
Age category	n	%	IR	n	%	IR	n	%	IR
Under 14	12	9.5	5.8	8	12.1	42.8	4	6.7	2.1
Under 16	28	22.2	5.1	13	19.7	25.5	15	25.0	3.0
Under 18	58	46.0	5.7	31	47.0	32.1	27	45.0	2.9
Senior team	28	22.2	2.7	14	21.2	18.8	14	23.3	1.5
Total population	126	100	4.5	66	100	27.4	60	100	2.3

IR = injury incidence rate, n = number of injuries.

### Functional ankle instability (FAI) and dynamic postural control

**Figure 1:**
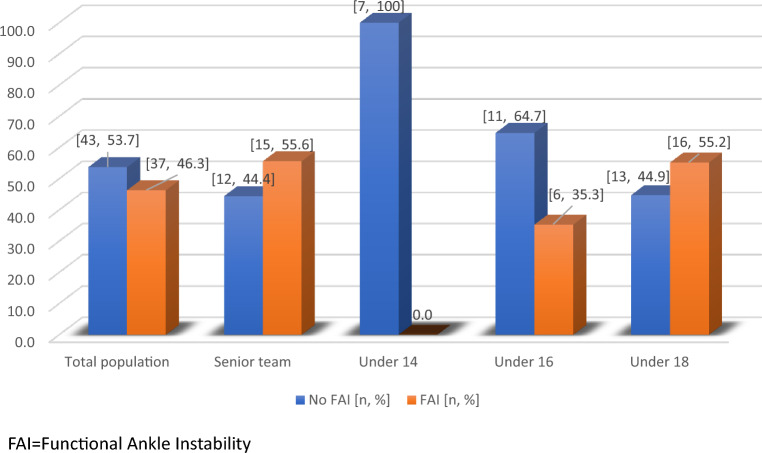
Self-reported functional ankle instability (FAI) among all players and per age categories per the Cumberland ankle instability test (CAIT).

Based on the CAIT questionnaire responses, 46.25% of the players (n = 37) reported having FAI. Figure 1 shows the self-reported presence and absence of FAI among players.

The mean composite score for the mean reach distances for the population according to the SEBT was 90.8 ± 6.7 (left limb) and 91.7 ± 6.8 (right limb). Table 3 shows the mean result for the SEBT composite s cores for the total population and age categories.

**Table 3:** Results of the SEBT for the total population and age categories.

	SEBT	Mean	SD	95% CI
	Lower limb	Composite scores		
Total population [n = 80]	Left	90.8	± 6.7	89.3–92.3
	Right	91.7	± 6.8	90.2–93.2
U 14 [n = 7]	Left	98.6	± 6.6	92.5–104.8
	Right	97.7	± 6.6	91.6–103.7
U 16 [n = 16]	Left	91.1	± 5.9	88.0–94.11
	Right	92.8	± 6.1	89.7–96.0
U18 [n = 29]	Left	89.1	± 5.8	86.9–91.3
	Right	89.6	± 5.7	87.5–91.8
Senior team [n = 27]	Left	90.4	± 6.8	87.8–93.2
	Right	91.7	± 7.6	88.8–94.7

CI = Confidence Interval, n = Number of participants, SD = Standard Deviation, SEBT = Star Excursion Balance Test.

### Ankle dorsiflexion (DF) range of motion (ROM)

The average ankle DF ROM for the left ankle joint was 10.35 ± 2.9 cm and right ankle joint was 10.48 ± 3.0 cm. Table 4 shows the ankle DF ROM for the total population, as well as, per age group.

**Table 4:** The ankle dorsiflexion (DF) range of motion (ROM) of football players at a football academy in Ghana for the total population and per age group.

	Ankle DF ROM	Mean	SD	95% CI
Total population (n = 80)	Left	10.4	2.9	9.7–11.0
	Right	10.5	3.0	9.8–11.1
Under 14 (n = 7)	Left	10.6	3.6	7.2–13.9
	Right	10.4	3.0	7.7–13.2
Under 16 (n = 17)	Left	10.3	3.6	8.4–12.2
	Right	10.8	3.5	9.0–12.6
Under 18 (n = 29)	Left	10.8	2.7	9.7–11.8
	Right	10.8	2.8	9.8–11.9
Senior team (n = 27)	Left	9.9	2.6	8.9–10.9
	Right	9.9	3.0	8.7–11.1

CI, confidence interval; DF, dorsiflexion; n = number of participants; ROM, range of motion; SD, standard deviation.

### Association between intrinsic factors and injury

The associations between the selected intrinsic factors and injury incidence were calculated using Spearman’s rank correlation and represented in Table 3. Age was negatively associated with both match (r = − 0.294, *p* = 0.008) and training injury incidence (r = − 0.314, *p* = 0.005), while BMI was associated with overall (r = − 0.513, *p* = 0.000) and training incidence (r = − 0.395, *p* = 0.000), and CAIT score was associated with match injury (r = − 0.244, *p* = 0.029). See Table 5 for details.

**Table 5:** Association between intrinsic factors and injury incidence among football players at a football academy in Ghana (n = 80).

	Overall injury incidence rate					Match injury incidence rate					Training injury incidence rate				
Variables	Total sample (n = 80)	U 14 (n = 7)	U 16 (n = 17)	U 18 (n = 29)	Senior team (n = 27)	Total	U 14 (n = 7)	U 16 (n = 17)	U 18 (n = 29)	Senior team (n = 27)	Total	U 14 (n = 7)	U 16 (n = 17)	U 18 (n = 29)	Senior team (n = 27)
						Sample (n = 80)					Sample (n = 80)				
Age	− 0.589**					− 0.294**					− 0.314**				
BMI	− 0.513**	0.292	− 0.295	− 0.428*	0.144	− 0.215	0.658	0.074	− 0.287	0.095	− 0.395**	− 0.655	− 0.42	− 0.279	0.057
Previous injury (yes)	0.133	0.171	0.338	0.453*	− 0.306	0.072	0.502	− 0.028	0.22	0.02	0.127	− 0.548	0.453	0.458*	− 0.259
Limb dominance (right)	− 0.001	− 0.22		− 0.141	− 0.021	− 0.156	0.108		− 0.188	− 0.337	0.08	− 0.354		0.097	0.227
Ankle DF ROM (left)	− 0.073	− 0.28	− 0.374	− 0.148	0.303	− 0.219	− 0.49	− 0.211	− 0.096	− 0.178	0.122	0.599	− 0.16	− 0.164	0.436*
Ankle DF ROM (right)	− 0.033	0.059	− 0.358	− 0.163	0.258	− 0.181	0.048	− 0.445	− 0.156	− 0.062	0.099	0.147	− 0.049	− 0.116	0.289
Left composite score (SEBT)	0.126	0.154	− 0.036	− 0.036	− 0.066	0.024	− 0.038	− 0.05	− 0.05	− 0.071	0.039	0.433	− 0.168	− 0.168	0.047
Right composite score (SEBT)	0.118	− 0.27	0.121	0.121	− 0.103	− 0.016	− 0.378	0.034	0.034	− 0.171	0.045	0.433	− 0.032	− 0.032	0.026
CAIT score	0.263*	0.241	0.123	0.123	0.071	0.244*	0.189	0.039	0.039	0.139	0.051	0	0.028	0.028	− 0.076

*BMI* body mass index, *CAIT* Cumberland ankle instability, *DF* dorsiflexion, *IR* injury incidence rate, *ROM* range of motion.

***p ≤ 0.001, **p ≤ 0.01, *p ≤ 0.05.

Significant values are in bold.

### Association between extrinsic factors and injury

The association between the selected extrinsic factors and injury incidence were calculated using the Spearman’s rank correlation, represented in Table 6.

**Table 6:** Association between extrinsic factors and injury incidence among football players at a football academy in Ghana (n = 80).

	Overall injury incidence				Match injury incidence					Training injury incidence				
Variables	Total sample (n = 80)	U 14 (n = 7)	U 16 (n = 17)	U 18 (n = 29)	Total sample (n = 80)	U 14 (n = 7)	U 16 (n = 17)	U 18 (n = 29)	Senior team (n = 27)	Total sample (n = 80)	U 14 (n = 7)	U 16 (n = 17)	U 18 (n = 29)	Senior team (n = 27)
Player position														
Goalkeeper	− 0.171		0.371	0.159	0.241*		0.272	− 0.363	− 0.131	− 0.01		− 0.237	0.173	0.12
Defender	− 0.021	− 0.171	− 0.038	− 0.231	0.085	− 0.502	0.263	− 0.054	0.367	− 0.122	0.548	− 0.21	− 0.312	− 0.12
Midfielder	− 0.058	0	− 0.113	0.056	− 0.044	0.229	0.136	0.052	− 0.349	− 0.084	− 0.417	− 0.237	0.087	0.024
Attacker	0.196	0.171	0.479	0.293	0.119	0.251	− 0.183	0.247	0.063	0.215	− 0.091	.669**	0.138	0.018
Member of a national team														
Yes				− 0.166				0.007	0.337				− 0.23	− 0.143
No	0.193			0.166	− 0.03			− 0.007	− 0.337	0.204			0.23	0.143
Exposure hours														
Total exposure	0.599**				0.302**					− 0.302**				
Match Exposure	− 0.192				− 0.176					− 0.037				
Training Exposure	0.599**				0.302**					− 0.302**				

BMI, body mass index; CAIT, Cumberland Ankle Instability; DF, dorsiflexion; IR, injury incidence rate; ROM, range of motion;

***p ≤ 0.001, **p ≤ 0.01, *p ≤ 0.0.

Significant values are in bold.

## Discussion

This may be the first prospective study to report on the risk factors associated with football injuries among elite youth and adult academy players in Ghana. The aim of the study was to determine risk factors such as age, BMI, limb dominance, previous injury, ankle DF ROM, SEBT scores, self-reported FAI, national team membership, player position and exposure hours associated with match and training injuries among Ghanaian male academy football players. The study found that age, BMI, and exposure hours were negatively associated with overall, match, and training injury incidence.

Controversy exists whether age is indeed an injury risk factor in football. While some studies have suggested that increased age is associated with injury among youth and adult players^27,28^, other studies have reported no evidence of such an association^29,30^. We observed a negative association between age and overall injury incidence, as well as, match and training injury incidence. In our study, younger players may tend to have a high injury incidence compared to older players, which corroborates a few other studies^31,32^. Younger players experience stronger forces and strain as their muscular systems expand in both length and size to compensate for their skeletal system’s faster growth at younger ages^33^. During this stage of maturation, high amounts of repetitive loading from training and matches are more likely to increase injury incidence^33^. Further studies should compare the relationship between biological and maturation age with injury prevalence and incidence.

Hägglund et al.^34^ found that neither height, weight, nor BMI was significantly associated with injury incidence among players with a mean age of 25 ± 5 years. Though preliminary, our findings are interesting as they contradict previous studies that found that higher BMI was associated with an increased incidence of injuries^30,35^. The reason for our finding is uncertain. However, a recent study among English Premier League professional footballers revealed that players with decreased BMI may be prone to greater injury burden^36^ which corroborates our finding. Our findings suggest that football academies should be aware that smaller or lighter players are at a higher risk of injury and that additional attention should be placed on screening and training loads, as well as injury prevention techniques.

Our findings revealed that U18 players with a previous injury in the last 12 months were at risk of sustaining an injury in training. Most risk factor studies^30,37–39^ corroborate our findings. Previous injury has also been reported as a risk factor among U12 and U18 players who experienced a previous injury presenting a double-fold risk of a second occurrence^40^. About 79% of the players in our study had sustained an injury in the past 12 months prior to the study, most of which were U18s, who also sustained 71.4% of recurrent injuries recorded. As a result of a first injury, there may be physiologic or skeletal alterations in the joints or muscles that make players more prone to re-injury^41^. This discovery emphasises the necessity of thorough injury history reporting during the initial screening of players and follow-up observation of changes in movement patterns following an injury. Additionally, to facilitate adequate neuromuscular control, proper rehabilitation and training treatments of players are needed.

Evidence suggests that it is important to keep track of the history of football-related injuries to identify at-risk players in male football population^37–39^. Injury prevention strategies might then be targeted to these players^37–39^. However, since a risk factor may not be the same as a predictive factor^42^, injury prevention programs such as neuromuscular training^43^ and proprioceptive training^44^ have been shown to benefit football players and should be made available to all players and not only players at risk. For instance, joint sprain was the most common injury in our study. To reduce the number of joint sprain injuries, there should be an initiative to incorporate strengthening exercises of muscles around the joint^45^ for all academy players (especially the knee and ankle).

Generally, the association between limb dominance and injury risk or injury rates is well known^27,28,46^. However, the literature is not clear as to the significance of limb dominance being a risk factor for suffering injuries, specifically ankle sprains or instability. Our findings suggest that limb dominance was not associated with injury incidence among Ghanaian players which is supported by one study^30^. The fact that many players place more stress on their dominant limb, especially during high-demand activities, results in increased frequency and magnitude of moments about the ankle, which is one of the main reasons why limb dominance has been implicated as a risk factor for lower extremity injuries^47,48^. A previous study indicated that football players aged 14 to 18 who were left-leg dominant tended to be at a higher risk of injury than those who were right-leg dominant^32^. The reasons for these discoveries are still speculative.

About 40% of football players exhibit functional ankle instability after lateral ankle sprains^49,50^. We found that approximately 46% of players presented with FAI per CAIT measurements. Hertel^50^ proposed that multiple sensory deficiencies brought on by ankle sprains can result in instability, which raises the possibility of subsequent sprains. Numerous studies have looked into this notion; however, the results are mixed^51,52^. In a study of Portuguese athletes, a significant portion of the participants displayed indicators of self-perceived instability in their dominant limbs with regard to injury rates^28^. In our study, there was a significant association between self-perceived FAI (measured by the CAIT) and overall injury incidence, as well as, match injury incidence but not with training injury incidence. Despite the paucity of studies on FAI, particularly in Africa, it is vital to appreciate the CAIT as an important marker of potential injury and should be used regularly as a screening tool during the season.

In our study, about 42.5% of players were at risk of injury per dynamic postural control on the SEBT. However, players’ dynamic postural control on the SEBT was not significantly associated with injury incidence in our study which is corroborated by one study conducted among female football players with an average age of 21 ± 4.0 years^53^. A previous study among college American football players showed an association between dynamic postural control and injury risks^54^. The difference in findings may be attributed to the type of sport as well as, the age differences of the study samples.

In most sports that involve movements in multiple directions, ankle DF ROM has a significant impact on change of direction and landing^55,56^. When the ankle DF ROM is limited, the stiffness of the lower limb and forces of landing after a jump are usually altered which has the tendency to raise the risk of injury^55,56^. We found a significant association between left ankle DF ROM and the incidence of injuries during training for players older than 17 years (senior team). Though our findings are preliminary, Bradley and Porta’s^57^ study supports our finding, where a relatively weak association was found between ankle DF ROM and ankle injury rates. However, Arnason et al.^58^ reported no association between ankle DF ROM and injury incidence as was observed among younger players in our study. The lack of significant association observed in our study might be due to the relatively small sample size. A larger sample size might have revealed significant correlations; our findings regarding ankle DF ROM are thus preliminary.

In our study, being a goalkeeper was weakly but significantly associated with the incidence of injury during matches, while being in the U16 attacker position was strongly associated with the incidence of injury. A possible explanation for this finding is that goalkeepers appear to be under higher expectations during matches, especially when their injuries can occasionally affect individual and team performance^59^. Young attackers may not be skilled enough and may lack the technique to protect themselves during training or matches, hence their higher injury rate. Contrary to these findings, some studies have reported that playing position is not associated with injury rates^60,61^. Investigations on playing position and injury rate have been constrained by a dearth of individual exposure documentation, and many of the available studies used small samples or only included match injuries; hence, there may be a possible discrepancy in findings^60–62^. However, even with the finest study design, it may be challenging to provide a straightforward and unambiguous message concerning playing position and injury risk due to the variety of playing styles and same players playing different positions in modern football^63^. The current study did not examine the relationship between playing position and specific injuries. Future research should concentrate on risk factor associations with specific injuries to give a more complete picture.

Interestingly, we found a negative association between exposure hours and injury incidence which means that players with less exposure hours reported higher injury rates. This could be explained by the fact that being under-loaded during games may act as a mediator for injury^64^. Moreno and colleagues^64^ found that players sustaining most muscle injuries typically displayed substantially less accumulated match playing exposure in a season, which corroborates our findings. Contrastingly, most studies have argued that the cumulative effect of repeated and prolonged exposure to football at the end of the season may increase the prevalence or incidence of injury^65,66^. Though a relationship between exposure hours and injury incidence has been widely reported, recorded injury incidences are different for every country^1,29^. For example, players from northern Europe have substantially greater total injury rates than those from southern Europe^67^ possibly due to the aggressive style of play among the Southern European football players. Although our findings showed a negative correlation between injury incidence and exposure hours, we observed a high total exposure for U18s (10 138 h) which is the stage for promotion to the senior team to start a professional career. This result can provide insight into the coaching staff's efforts to get players physically ready for the demands of professional football.

Being a member of a national team was not associated with injury incidence in our study. Only nine of the 80 players in our study were members of the national team. The small number of players who participated for their national teams may explain this finding. A retrospective study in adult female football players^68^, found a significant association between players who were members of their national team and increased injury rates. The studies attributed their finding to possible higher exposure to football when part of a national team as well as the level of competitiveness and the desire to stay in the national team.

Although our study is the first of its kind in Ghana, the small sample size is a potential weakness and thus limits the generalisability of the findings. Another problem with sample size was also evident when finding the associations of selected factors and injury rates for the age categories where injury cases were small. As suggested by Bahr and Holme^69^, about 20–50 injury cases are required to detect moderate to strong associations in a risk factor study, whereas small to moderate associations would need about 200 injured subjects. Our study recorded 128 injury cases for the combined sample size and 12, 28, 58 28 injury cases for U14, U16, U18 and senior players, respectively. Due to this limitation, we urge using caution when interpreting the results, particularly with regard to their generalizability, as our findings are preliminary. Our research cannot give a complete account of the injury risk among male academy football players since injuries are likely the result of a complex interaction of several factors, all of which were not investigated in this study. The current study could not investigate the influence of match and training loads, the playing surface, and weather conditions, which we acknowledge may have influenced the injuries sustained. One strength of the study was that it relied on prospectively recorded data avoiding the risk of recall bias. Another strength was recording individual player exposure hours during training and matches, which allowed for risk factor analysis.

## Conclusion

The study identified possible risk factors associated with injury among both young and adult academy players. Specifically, the study found an association between overall, match or training injury incidence and age, BMI, previous injury, goalkeeper and attacker position, ankle DF ROM, and self-perceived FAI. These results offer preliminary evidence for further research encompassing other risk factors and multivariate analyses, as well as for the creation of practical and pertinent preventive measures tailored specifically for the population of football academies. Further studies should focus on examining risk factors for specific anatomical locations, especially the ankle and the knee as well as specific injury types among players using a larger sample size.

## Data Availability

The datasets used and/or analysed during the current study are available from the corresponding author on reasonable request.
